# The Psychology of Tuberculosis

**Published:** 1904-02-13

**Authors:** 


### The Psychology of Tuberculosis.

The physician and the novelist, and, indeed, almost all serious students of the human, have been fascinated by the peculiar attractiveness of a study of the influence of morbid affections of the body on mental and moral processes, and to many the subject of the psychology of tuberculosis, as it may conveniently be termed, is peculiarly rich in suggestiveness. The importance of considering the temperament of the phthisical patient as an ætiological factor is no longer insisted upon, and there is, indeed, some danger in these days when hygienic measures dominate thought and guide action that the mental and even purely emotional aspects may too readily be relegated to the background. The psychopathology of tuberculosis has long been a favourite subject for portrayal in fiction. The dramatist, the novelist, and the poet have all been attracted by the pathos which surrounds the decline of the phthisical, and have sought to present the pathetic features of life's slow ebbing linked with the, in only too many cases, hopelessness of improvement, and a despairing tenacity that clutches at a flickering vitality and thrusts Death's cold hand aside. In many modern works, however, the simple delineation of the clinical features is wrapt around with subtle analysis of psychological problems, perplexities of temperament, and puzzles of environment.

No one can have watched the life of a patient at a sanatorium for consumption without having realised the importance of not neglecting the moral and mental control, as well as securing the physical restoration. A recent French novelist has attempted to depict certain traits of the consumptive which at least in their tendencies must be considered immoral. Much in the life of the phthisical invalid tends to develop a peculiarly irritating egoism; and many features of institutional treatment of consumption undoubtedly go far to encourage selfishness, and perhaps in some instances foster erotic as well as other neurotic inclinations. It is, of course, well recognised that tuberculosis may bring about such mental derangement as may lead the sufferer into insane acts, or plunge him among criminal transgressors, and even where there is no rapidly developed or marked psychological alteration, a slow change in what we are pleased to call character undoubtedly ensues in a large number of cases, and is most noticeable in the slow development of a loss of will power, or undermining of the elements of self control, the ascendency of selfishness, and an abnormally increased susceptibility to external influences. There is also often extreme variability of temper, and as the case advances the ready development of fatigue after mental or physical exertion. The bearing of all this on treatment is manifest. The best physician and the most successful nurse must knowingly, or by natural tact and intuition, meet the special psychological needs of the consumptive with judgment and wisely-tempered personal control. In seeking to clear up the many problems which still surround and perplex the pathology of tuberculosis, we cannot afford to neglect to secure an intimate insight into the mysterious meanderings of the psychology of the subject.

